# Falsely Elevated 25-Hydroxy-Vitamin D Levels in Patients with Hypercalcemia

**DOI:** 10.1155/2020/8873506

**Published:** 2020-08-09

**Authors:** Moon Kyung Choi, Prapaipan Putthapiban, Patamaporn Lekprasert

**Affiliations:** ^1^Department of Internal Medicine, Einstein Medical Center Philadelphia, 5501 Old York Road, Philadelphia, PA 19141, USA; ^2^Department of Endocrinology, Einstein Endocrine Associates, 50 East Township Line Road Medical Arts Building, Suite G01, Elkins Park, PA 19027, USA

## Abstract

Symptomatic hypercalcemia is a commonly encountered clinical scenario. Though it is important to collect detailed history to find clinical clues connecting to the etiology of hypercalcemia, the diagnostic workup of hypercalcemia depends heavily on laboratory analysis. Accurate measurement of the parathyroid hormone and vitamin D levels is essential. However, commercial laboratory measurement of vitamin D levels can be erroneous in the setting of abundant paraprotein in the serum. One of the most common conditions that can cause an increased amount of paraproteins is multiple myeloma. We report 2 cases of falsely elevated 25-hydroxy-vitamin D levels in patients presenting with hypercalcemia and an underlying diagnosis of MM.

## 1. Introduction

Hypercalcemia is a well-established complication in patients with multiple myeloma (MM). Hypercalcemia due to MM is mediated by increased production of osteoclast-activating paracrine molecules by the tumor cells. Even in patients with underlying MM who present with hypercalcemia, measurement of the parathyroid hormone (PTH) and 25-hydroxy-vitamin D (25 (OH)-vit D) levels is integral for complete diagnostic investigation. Unfortunately, commercial laboratory analysis of the 25-hydroxy-vitamin D level carries limitations. This possibility of laboratory error is often overlooked by clinicians which may lead to invalid interpretation of laboratory results. Here, we describe 2 cases of erroneously elevated 25-hydroxy-vitamin D levels in MM patients presenting with symptomatic hypercalcemia.

## 2. Case Presentation

### 2.1. Case 1

A 65-year-old woman with a past medical history of hypertension and MM presented with fatigue. She was not started on treatment for MM except for recent radiation therapy to a metastatic lesion in the thoracic spine. Laboratory analysis was notable for chronic anemia (hemoglobin 7.0 g/dL, normal 12–16 g/dL), acute kidney injury (creatinine 3.1 mg/dL, normal 0.6–1.0 mg/dL), and serum calcium level of 16.6 mg/dL (corrected calcium 18.0 mg/dL, normal 8.4–10.3 mg/dL). Total protein level was elevated to 11.1 g/dL (normal 6–8.3 g/dL), and gamma globulin level was 6.0 g/dL (normal 0.6–1.6 g/dL). As there was no worsening of patient's anemia from her personal baseline, her fatigue was attributed to hypercalcemia. Further blood work revealed a PTH level of 8.0 pg/mL (normal 9–73 pg/mL). 25 (OH)-vit D level was >96.0 ng/mL via immunoassay with a reference normal range of 30 to 50 ng/mL. 1,25-Dihyroxy-vitamin D level was 12 pg/mL (normal 18–72 pg/mL). PTH-related protein (PTHrP) level was 12 pg/mL (normal 14–27 pg/mL). These laboratory findings indicated that the patient had PTH and PTHrP-independent hypercalcemia. The elevated 25 (OH)-vit D level suggested vitamin D toxicity. However, the patient was not on any vitamin D supplements, and the clinical suspicion for vitamin D intoxication was very low. To evaluate for laboratory error, 25 (OH)-vit D level was measured via liquid chromatography-tandem mass spectrometry (LC-MS/MS) which revealed a normal value of 46 ng/mL (normal 30–100 ng/mL). Based on low PTH level, normal 25 (OH)-vit D level, low 1,25-dihydroxy-vitamin D level, and low PTHrP level, the patient was diagnosed with hypercalcemia secondary to multiple myeloma. Her hypercalcemia was treated with intravenous fluids, calcitonin, and zoledronic acid. The patient's hypercalcemia showed a good response to treatment in the following days.

### 2.2. Case 2

A 72-year-old man with MM receiving melphalan and dexamethasone for palliation presented with confusion. His other comorbidities were hypertension, paroxysmal atrial fibrillation, chronic systolic heart failure, and a history of ischemic stroke. The family provided medical history on the patient's behalf. The patient had complained of thirst few hours ago and then became disoriented. Family members have not observed any focal weakness or dysarthria. The patient had not mentioned chest pain or shortness of breath. Initial blood work showed chronic anemia (hemoglobin 7.7 g/dL), acute kidney injury (creatinine 1.3 mg/dL), and hypercalcemia 14.9 mg/dL (corrected calcium 16.1 mg/dL). Total protein level was increased to 12.3 g/dL. A CT head scan did not reveal any acute disease process. Further investigation revealed a PTH level of 16.2 pg/mL and 25 (OH)-vit D level >96.0 ng/mL via immunoassay. 1,25-Dihyroxy-vitamin D and PTHrP levels were not measured. Similar to the previous case, the patient was not on any vitamin D supplements, and this raised concerns of an erroneous measurement of the 25 (OH)-vit D level. 25 (OH)-vit D level was measured again by LC-MS/MS and was found to be in the normal range (68 ng/mL). Normal PTH and 25 (OH)-vit D levels supported the high clinical suspicion for hypercalcemia secondary to MM. The patient's hypercalcemia improved when treated with intravenous fluid, calcitonin, and zoledronic acid. He was also continued on a palliative regimen for MM including dexamethasone. The patient's hypercalcemia and disorientation improved, and he was safely discharged to a nursing facility.

## 3. Discussion

25 (OH)-vit D level is widely accepted as a standard value representing the vitamin D status of a patient. Various methods are utilized to measure 25 (OH)-vit D levels such as automated immunoassay, competitive protein-binding assay, and LC-MS/MS [1]. Among these different methods, automated immunoassay is the most commonly used technique in commercial laboratories [2].

The initial measurements of the 25 (OH)-vit D level for the above 2 cases were carried out by Abbott Architect i2000 automated immunoassay. In the Architect i2000 immunoassay method, sheep polyclonal anti-vitamin D IgG is incubated with patient's serum. After sheep polyclonal anti-vitamin D IgG binds to 25 (OH)-vit D in the sample, chemiluminescence-labeled 25-hydroxy-vitamin D is added. Excess sheep polyclonal anti-vitamin D IgG that was not bound to 25 (OH)-vit D in the sample will bind to this exogenous vitamin D. After washing, only sheep anti-vitamin D IgG that is bound to exogenous vitamin D will emit chemiluminescence. Thus, the amount of chemiluminescence will be inversely associated with the quantity of 25 (OH)-vit D in the sample.

The repeat measurements of 25 (OH)-vit D levels were carried out by the AB SCIEX 6500 LC-MS/MS system. LC-MS/MS is largely accepted as the most accurate method of measuring 25 (OH)-vit D levels [1]. During LC-MS/MS, 25 (OH)-vit D is separated from other potential interfering molecules via chromatography. We hypothesize that the abundant paraprotein from MM may have interfered with the binding of sheep polyclonal anti-vitamin D IgG in the immunoassay. The abundant paraprotein may have had cross-reactivity to sheep polyclonal anti-vitamin D IgG, thus decreasing the amount of chemiluminescence. Total protein level was elevated in both cases, pointing to the abundance of the paraprotein from underlying MM. Cross-reactivity is a well-described interference in all immunoassays regardless of the measured molecule [3]. Further studies are required to investigate whether the concentration and cross-reactivity of paraproteins both affect the likelihood of laboratory error in 25 (OH)-vit D quantification.

We found 2 previous case reports of laboratory interference in the immunoassay measurement of 25 (OH)-vit D levels [2, 4]. Similar to our cases presented here, Ong et al. suspected that the falsely elevated measure of 25 (OH)-vit D was caused by an increased number of immunoglobulins from MM. In the second case report, immunoglobulins due to MM and rheumatoid factor were both considered as possible interfering molecules. Other serum molecules known to interfere with the 25 (OH)-vit D immunoassay include bilirubin, triglyceride, and anti-animal antibodies [2]. Our 2 patients did not have rheumatologic disease or hypertriglyceridemia. Total bilirubin levels were also normal in both patients.

In conclusion, it is important for clinicians to be aware of the limitations of the immunoassay measurement of the 25 (OH)-vit D level in the setting of MM. Careful interpretation of laboratory data in correlation with clinical manifestation is important. When there is discordance between laboratory data and clinical clues, close collaboration with the laboratory is crucial to perform analysis with alternative methods and evaluate for laboratory interference.
